# Mesenchymal stem cells in breast cancer: response to chemical and mechanical stimuli

**DOI:** 10.18632/oncoscience.381

**Published:** 2017-11-28

**Authors:** Seiichiro Ishihara, Suzanne M. Ponik, Hisashi Haga

**Affiliations:** Faculty of Advanced Life Science, Hokkaido University, Kita-ku, Sapporo 060–0810, Japan

**Keywords:** mesenchymal stem cells (MSCs), cancer associated fibroblasts (CAFs), tumor microenvironments, mechanical stimuli, prosaposin

The tumor microenvironment contains not only cancer cells but also non-cancerous cells and extracellular matrices (ECMs). These cell types interact through chemical and mechanical stimuli and as a result, regulate tumor progression. While many studies have identified chemical stimuli that are critical for cancer progression, the role of mechanical stimuli is less defined. It has been recently shown that mechanical stimuli, especially the stiffness of ECMs, contribute to tumor progression in some specific cancers including breast, colorectal, and lung [1, 2, 3]. In particular, cancer cells respond to stiffness of surrounding ECMs and modulate their malignant phenotypes. However, the specific cell types and mechanisms of cancer progression regulated by ECM stiffness are not completely understood.

Mesenchymal stem cells (MSCs) are multi- potent cells existing in major types of connective tissue which have recently been shown to migrate to tumor sites and mediate tumor growth and progression [4]. In response to chemical stimuli, MSCs are recruited to the tumor microenvironment where they can differentiate into cancer associated fibroblasts (CAFs) and support tumor progression. Emerging evidence continues to reveal multiple functions for MSCs within the tumor microenvironment, however, we are the first to show that mechanical stimuli in the tumor microenvironment contributes to the differentiation of MSCs to CAFs.

**Figure 1:**
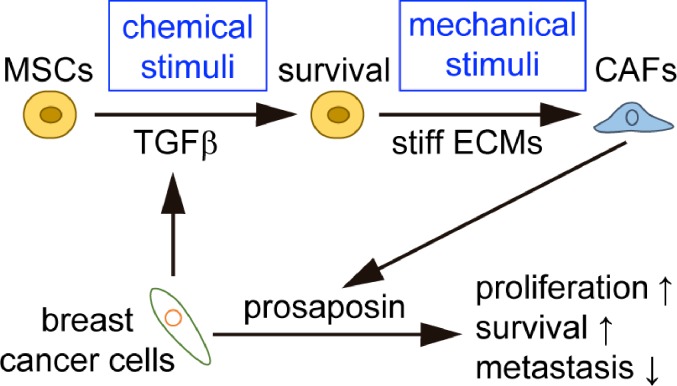
Mesenchymal stem cells (MSCs) respond to chemical and mechanical stimuli in breast cancer TGFβ: transforming growth factor β, ECMs: extracellular matrices, CAFs: cancer associated fibroblasts.

Recently we reported that MSCs respond to both chemical and mechanical stimuli in the mammary tumor microenvironment by promoting tumor progression [5]. Treating MSCs with conditioned media from breast cancer cells mimicked chemical stimuli and increased the survival rate of MSCs compared to control conditions. Transforming growth factor β (TGFβ) was identified as a key factor in conditioned media capable of inducing MSC survival (Figure 1). Importantly, we cultured MSCs on stiff or soft ECMs and investigated the differentiation of MSCs to CAFs. MSCs on stiff, but not soft, ECMs showed CAF-like phenotypes confirmed by morphology and expression of alpha smooth muscle actin, a marker of CAFs. Thus, we found that MSCs differentiate into CAFs in response to the mechanical stimuli of ECM stiffness (Figure 1). Furthermore, our results demonstrated that a combination of chemical and mechanical cues activate MSCs to promote tumor growth. Treatment of MSCs on a stiff matrix with tumor cell-conditioned media resulted in the secretion of prosaposin. This soluble factor promoted proliferation, survival and malignant phenotypes of breast cancer cells *in vitro* and *in vivo*. However, we found that prosaposin secreted from MSCs inhibited lung metastasis, while prosaposin knockdown in MSCs increased metastasis. Thus, prosaposin secreted from MSCs has contradicting roles for cancer progression: promoting cancer progression by enhancement of proliferation and survival whereas preventing cancer progression by inhibition of metastasis (Figure 1).

Interestingly, soluble factors such as vascular endothelial growth factor, hepatocyte growth factor, and TGFβ, reported to be secreted from CAFs [6], were not detected in the proteomics analysis of secreted factors from MSCs on stiff ECMs. This result indicated that the versatility of CAFs is dependent on their origins. One subset of CAFs are derived from MSCs while others originate from regional fibroblasts. This difference may contribute to the variable secretion profiles of CAFs and also the discrepancy in reports indicating a positive or negative contribution of CAFs in cancer progression.

To our knowledge, this is the first report to demonstrate that the mechanical stimulus of ECM stiffness and the chemical cue of TGFβ both play a critical role in the activation of MSCs and the promotion of cancer progression. These findings are critical for the field because they provide evidence that the stiffness and composition of the tumor microenvironment are critical determinants of cancer progression. In fact, the stiffness of the extracellular matrix may be the driving factor of chemical secretion that is necessary to differentiate and advance tumors toward metastasis.

These results are consistent with previous reports which showed that prosaposin drives tumor growth [7] and prevents metastasis in breast cancer [8]. We also found that high expression levels of prosaposin correlated with poor prognosis in grade 1 breast cancer patients while in contrast, good prognosis in grade 3 breast cancer patients. Grade 1 breast cancer is less metastatic than grade 3. Therefore, prosaposin may promote progression of grade 1 breast cancer by inducing proliferation and survival whereas prevent progression of grade 3 breast cancer by inhibiting metastasis. Prosaposin may be a useful therapeutic target for patients with the appropriate grad of breast cancer.
